# Exploring the benefits of wild plants in dietary nutrition: investigating perspectives, choices, health impacts and sustainable practices

**DOI:** 10.1186/s12906-024-04379-4

**Published:** 2024-02-14

**Authors:** Tauseef Anwar, Huma Qureshi, Sumbal Shahzadi, Ejaz Hussain Siddiqi, Hayssam M. Ali, Mohamed M. A. Abdelhamid, Muhammad Nazim

**Affiliations:** 1 Department of Botany, The Islamia University of Bahawalpur, Bahawalpur, 63100 Pakistan; 2 Department of Botany, University of Chakwal, Chakwal, 48800 Pakistan; 3 Department of Botany, University of Gujrat, Gujrat, 50700 Pakistan; 4 Department of Botany and Microbiology, College of Science, King Saud University, 11451 Riyadh, Saudi Arabia; 5 Agricultural Botany Department, Faculty of Agriculture (Saba Basha), Alexandria University, Alexandria, Egypt; 6 State Key Laboratory of Desert and Oasis Ecology, Key Laboratory of Ecological Safety and Sustainable Development in Arid Lands, Xinjiang Institute of Ecology and Geography, Chinese Academy of Sciences, Urumqi, 830011 PR China

**Keywords:** Traditional medicine, Ethnobotanical knowledge, Local food practices, Nutritional ethnobotany, Dietary benefits

## Abstract

**Background:**

This ethnobotanical study in Dunyapur, District Lodhran, Pakistan, focuses on traditional medicinal knowledge, exploring 41 plants across 28 families. The research involves 496 informants from diverse backgrounds, including farmers, herbalists, housewives, teachers, and shopkeepers. The prevalence of herbs (68%) aligns with their accessibility and rapid regrowth, shaping the local medicinal landscape. The study investigates socio-demographic features, emphasizing the importance of considering the community's diverse perspectives.

**Methods:**

The research employs quantitative ethnobotanical data analysis, introducing various indices like PPV, FUV, FIV, RFC, UV, and RI. The analysis of plant growth habits underscores the dominance of herbs, and the method of preparation evaluation identifies decoction as the most common (23%). Leaves (27%) are the most utilized plant part, and Resedaceae stands out with the highest FUV (0.38). FIV highlights the ecological and cultural significance of Poaceae, Boraginaceae, Fabaceae, and Solanaceae.

**Results:**

The RFC values range from 0.016 to 0.032, with Cucumis melo having the highest value (0.032), indicating its frequent citation and cultural significance. The study reveals specific plants like *Melia azedarach, Peganum harmala* and *Salvadora oleoides* with high PR values for skin issues, reflecting their widespread acceptance and effectiveness. *Oligomeris linifolia* emerges with the highest UV (0.38), emphasizing its greater significance in local traditional practices. *Leptadenia pyrotechnica* records the highest RI (9.85), underlining its exceptional importance in the community's traditional pharmacopeia.

**Conclusion:**

The findings offer a holistic understanding of ethnobotanical knowledge in Dunyapur, emphasizing the role of local contexts and ecological factors in shaping traditional plant uses. The study contributes valuable insights into the diverse practices within the community, laying the foundation for sustainable integration of traditional knowledge into broader healthcare frameworks.

**Supplementary Information:**

The online version contains supplementary material available at 10.1186/s12906-024-04379-4.

## Background

Ethnobotany investigates the interactions between humans and plants. Studies in ethnobotany encompass diverse connections between people and plants including their magical, religious, and therapeutic roles. Wild plants, a rich source of nutritional benefits significantly contribute to global food security. The increasing global awareness of the intrinsic value of these plants influenced by cultural perspectives and dietary choices highlights their potential to combat malnutrition, enhance diet diversity, and promote health. Embracing sustainable practices, wild plants emerge as a solution, fostering both ecological equilibrium and human well-being. Harnessing their capabilities holds the power to revolutionize worldwide nutrition and health environments [1].

Wild plants not only enrich global food diversity with their nutritional benefits but also promote eco-friendly practices, alleviating the strain on over-farmed lands. Embedded in various cultures as traditional dietary staples, these plants align with local food perceptions and preferences. Addressing malnutrition by offering essential micronutrients absent in processed foods, they exhibit resilience against climatic fluctuations. Understanding and promoting their nutritional advantages, aligned with sustainable practices and local preferences holds the key to enhancing global food security while preserving health and ecological balance [1, 2].

The utilization of herbal remedies extracted from plants has a historical presence across diverse cultures. Despite the decline in local understanding of therapeutic and wild food herbs worldwide, plants continue to play a crucial role in our daily existence. Since the inception of medicine, natural products, especially plant-derived ones, have supported human health. The pharmaceutical industry recognizes plants with a long history of ethnomedicine use as a valuable source of drugs. Ethnobotany investigates the use of therapeutic plants in local contexts, particularly in regions where herbal remedies often considered safer and healthier persist. As the world increasingly adopts sustainable practices the significance of wild plants grows offering a bridge between ecological balance and human well-being [3–6].

The utilization of plants as medicine holds global significance for public health. Medicinal plants have been a part of traditional healthcare, providing solutions for various needs and promoting overall wellness. Phytotherapy, the use of herbs in therapeutic procedures demonstrates efficacy in treating infectious disorders. Ethnobotanical surveys play a crucial role in understanding the relationship between communities and wild species aiding in the identification of new medications. In many remote and underdeveloped regions, the primary healthcare system relies significantly on medicinal plants, emphasizing their invaluable role as a natural resource [7].

Ethnomedicine explores how different cultures perceive health, illness, and disease influencing how people seek care and engage in healing rituals. Plants are indispensable for the survival of all species including humans, serving as major producers and transmitters on Earth. The intricate chemicals in therapeutic herbs contribute to the food, cosmetics, and medical industries. Herbal-based medications have made substantial contributions to human health providing sources for modern medications and prescriptions [8].

The World Health Organization defines medicine originating from plants as the sum of knowledge, skills, and practices based on indigenous theories and experiences. The scientific community's recent interest in the therapeutic potential of medicinal plants has historical roots dating back to the 1950s. Traditional medicine remains a primary treatment for approximately 70% of people in poor nations indirectly impacting industrialized countries relying on medicinal plants for pharmaceutical products. Traditional healers continue to play a vital role in rural communities preserving traditional knowledge and contributing to regional health structures [9–11].

Approximately 80% of the world's population uses herbs for treating illnesses and maintaining health. Mountainous and rural populations heavily depend on herbal flora for nutritional and food needs. Medicinal herbs are crucial for various medical conditions including heart and hepatic diseases, microbial infections, and non-communicable diseases like diabetes mellitus. Ongoing scientific efforts aim to develop potent antibacterial drugs with fewer adverse effects from these biofilm infections [12].

**Fig. 1:**
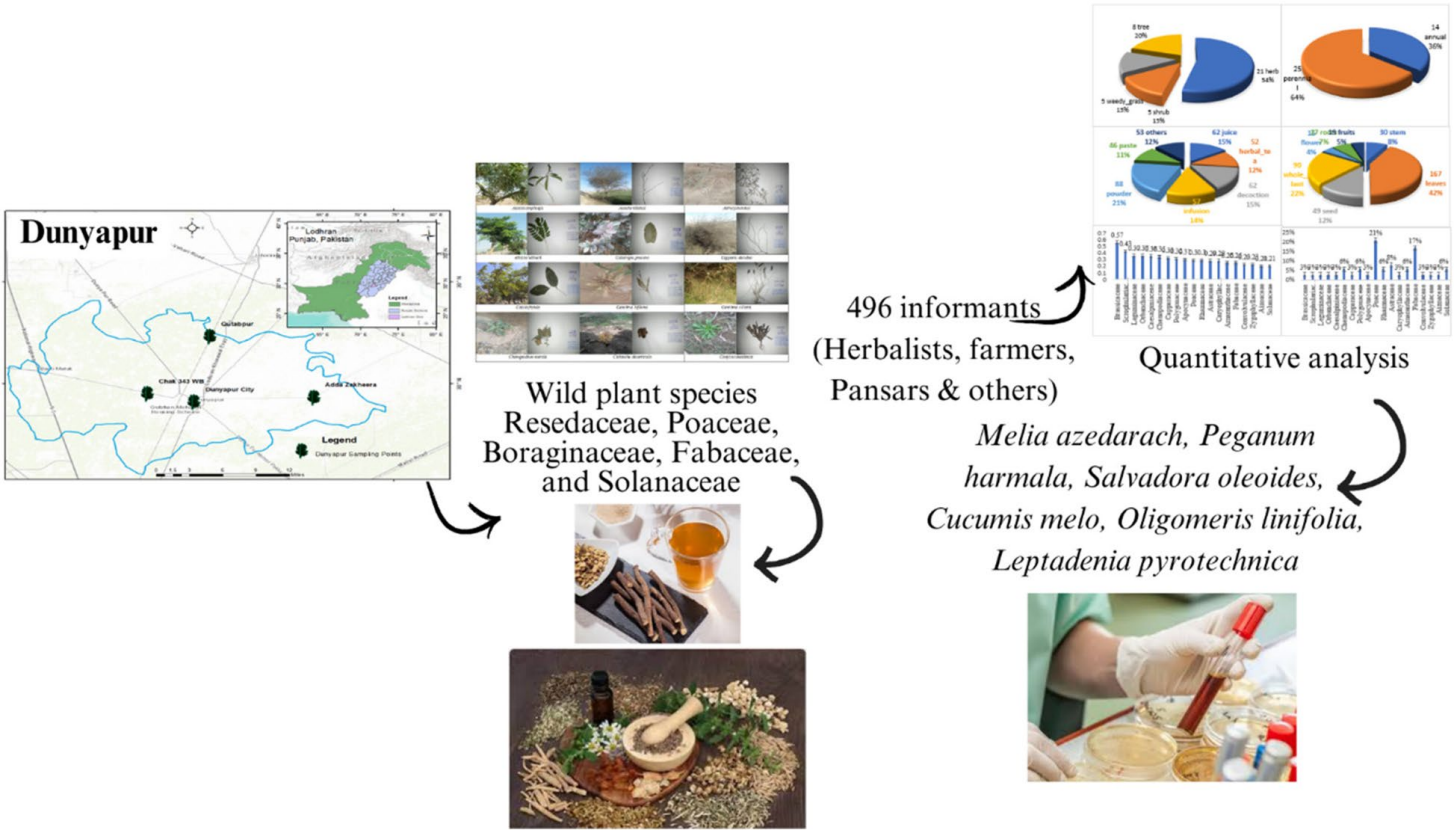
Exploring the Benefits of Wild Plants in Dietary Nutrition

Pakistan's diverse topography encompassing mountains, plains, beaches, and deserts hosts a wide variety of medicinal plants impacting the lives of its residents. Ethnopharmacological data on plants used for pediatric diseases offer valuable insights. An ethnobotanical survey in Dunyapur, District Lodhran, Pakistan highlights the importance of traditional wild flora and their role in local health care (Fig. 1). The study aims to identify and explore plants used in ethnomedicine employing quantitative analysis through ethnobotanical techniques.

## Materials and methods

Several surveys were undertaken to gather indigenous communities' traditional knowledge regarding the usage of medicinal plants in Lodhran. The aim was to investigate and compile information on ethnomedicinal flora within the study region.

### Study area

**Fig. 2:**
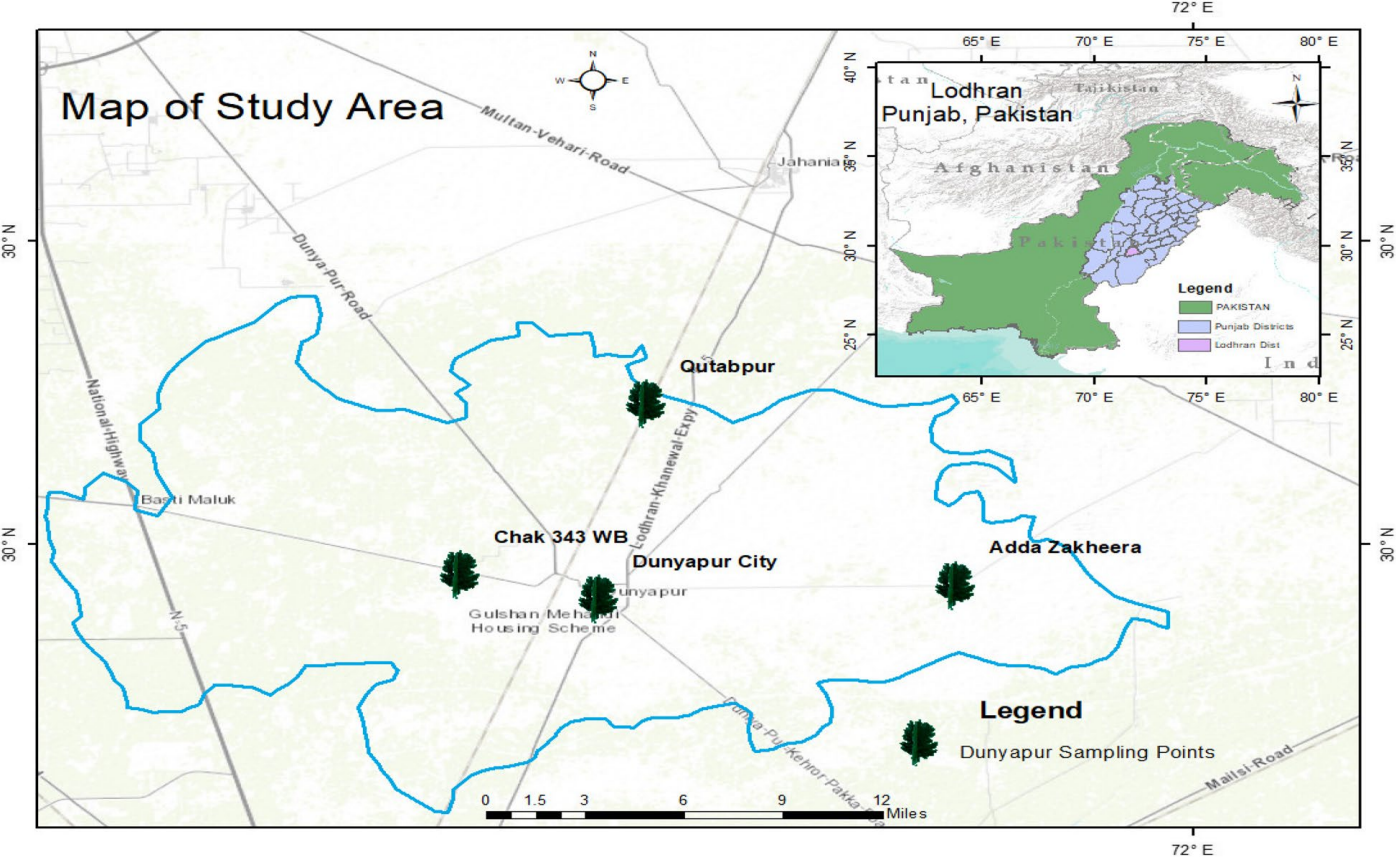
Map of the study area

**Fig. 3:**
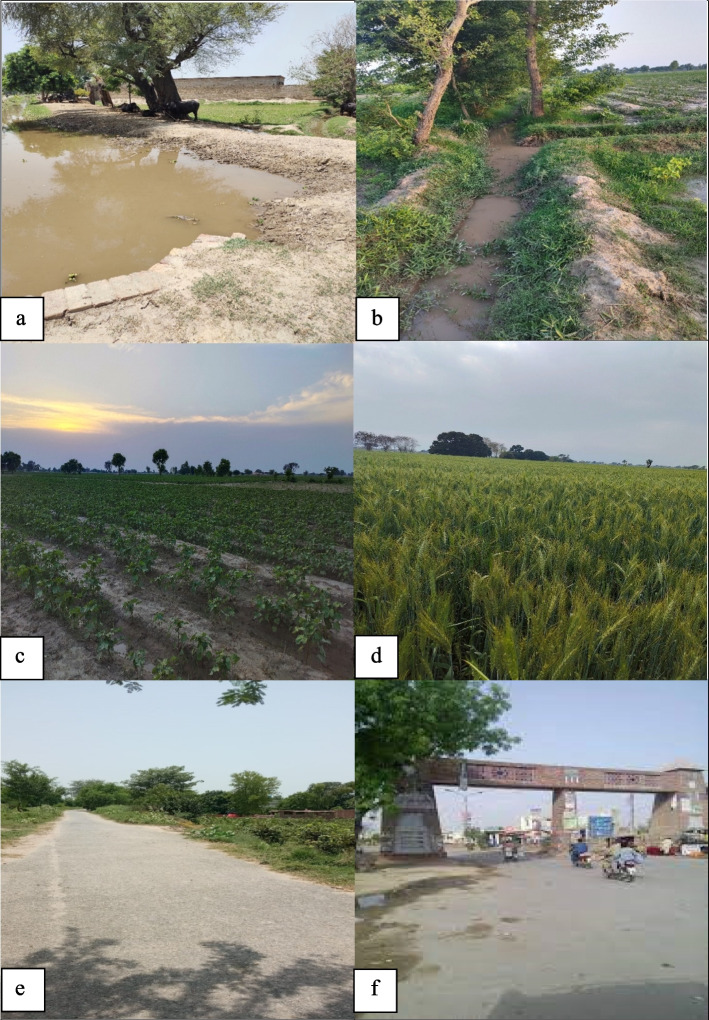
Sceneries of study area (**a**) Chak 343 W.B (**b**) Chak 309 W.B (**c**) Qutabpur (**d**) Adda Zakheera (**e**) Darbar Sultan Ayub (**f**) Dunyapur city

Dunyapur is located in District Lodhran, Punjab, Pakistan situated on the northern side of the river Sutlej. Its coordinates are approximately 27.70° North latitude and 68.86° East longitude. Map of the study area is presented in Fig. 2. The tehsil is characterized by a flat landscape extensively covered by canals and tube wells for irrigation purposes. The region's reliance on canals for irrigation and its distinct seasonal patterns contribute to the unique ecological dynamics of Dunyapur. Sceneries of study area including Chak 343 W.B, Chak 309 W.B, Qutabpur, Adda Zakheera, Darbar Sultan Ayub and Dunyapur city are shown in Fig. 3. The groundwater in the area is deemed palatable. Dominant vegetation includes dry deciduous forests and shrublands. Dunyapur experiences diverse climatic conditions with cold winters and hot, dry summers. The region encounters high temperatures from May to July. On average, the annual rainfall in Dunyapur is around 71 millimeters.

The education status of Dunyapur encompasses a range of educational institutions, from primary schools to higher education facilities, contributing to the overall literacy and knowledge base of the community. Access to healthcare and education, coupled with the preservation of cultural practices, reflects the multifaceted dynamics of this region.

Given its geographical features and climatic conditions, Dunyapur holds significance for ethnobotanical studies and research, particularly in understanding the traditional use of wild flora and its impact on the local ecosystem.

### Ethno-botanical survey and data collection

The initial research objective involved the collection and identification of wild flora in Dunyapur with strict adherence to the ISE code of ethics (http://ethnobiology.net/code-of-ethics/) during data gathering. Direct interviews were conducted using a structured questionnaire, supported by the Rapid Appraisal Approach (PRA). Introducing ourselves as the researchers, we established a brief rapport with respondents to ensure their confidence before commencing interviews. Given that a majority of informants, including farmers, housewives, and hakims did not converse in English, a questionnaire was translated into their local language (Punjabi), and their responses were then documented. The information provided by locals encompassed details such as local names, recipes, local usage, the plant part utilized, and their participation in data collection. For each plant species with therapeutic properties, the local names and growth characteristics were recorded. Following each interview, a systematic process of numbering, pressing, and drying sample specimens was executed to facilitate subsequent identification, utilizing the Pakistan e-flora (http://www.tropicos.org/Project/Pakistan) as a valuable reference source.

### Methods of quantitative ethnomedicinal data analysis

The assessment of the data involved the application of descriptive statistics, as well as qualitative and quantitative analysis methods. Ethnobotanical data were examined using Microsoft Excel spreadsheet software version 2016 and SPSS version 25.

### Family Importance Value (FIV)

To evaluate the relative significance of families, the FIV was used. It was computed by dividing the number of informants who mentioned the family by the total number of informants [13].

$${\text{FIV}}=\frac{\mathrm{FC }\left({\text{family}}\right) }{{\text{N}}}\times 100$$

Where “FC” is the number of informers revealing the family, while “N” is the total number of informants who participated in the research.

### Popular therapeutic use value (POPUT)

POPUT is a metric used to calculate the importance of a plant species for medicinal and therapeutic uses. Using the following formula, values for POPUT were determined [14].

$${\text{POPUT}}= \frac{{\text{NURIT}}}{{\text{TUR}}}$$

"NURIT" refers to the number of use reports for each sickness or treatment effect. “TUR” is the total number of use reports.

### Informant consensus factor (ICF)

The ICF was developed as a result of the search for the informant’s agreement on the stated treatment for each category of disease and determined by the given formula [15].

$${\text{ICF}}=\frac{\mathrm{Nur }-\mathrm{ Nt}}{\mathrm{Nur }- 1}$$

Where “Nur” explains the total use report for each category, while “Nt” explains the taxonomic numbers used. An estimate of the significance of each plant taxon in conventional knowledge is provided by consensus information-gathering methodology. The informant's agreement with the species that are used to cure a specific ailment falls under the rank of high ICF.

### Plant part value (PPV)

Plant part value is a measure of the relative importance of different plant parts in traditional medicines. The PPV of a plant species can vary depending on the cultural context and the specific needs of the community. It is computed what proportion of the plant's components such as its root, seed, leaves, flower, fruit, etc. are used [15].

$$\mathrm{PPV }\left(\mathrm{\%}\right)=\frac{\varepsilon RU(plant part)}{\varepsilon RU}\times 100$$

Where $\in {\text{RU}}\left(\mathrm{plant part}\right)=\mathrm{the sum}$ of uses reported per part of the plant and $\in {\text{RU}}=$ total number of uses reported of all parts of the plant.

### Relative frequency citation (RFC)

The formula below was used to calculate the index of the relative frequency of citations [15].

$${\text{RFC}}=\frac{{\text{FC}}}{{\text{N}}}$$

Where FC is the number of informants who reported using a species and N is the total number of informants.

### Use value (UV) of plant species

It is the quantitative measure of the relative significance of locally known plant species and was determined using the given formula [15].

$$\mathrm{UV }= \sum \frac{{\text{Ui}}}{{\text{N}}}$$

Where "UV" denotes each species' use value, "Ui" indicates the number of uses recorded by each informant for a specific species, and "N" is the total number of informants.

### Relative Importance (RI)

According to their relative importance (RI), each plant species' use and the body organ systems it treats are rated [16]. It is determined as.

$$\mathrm{RI }=\frac{(R.Ph + R.BS) }{2}\times 100$$

Where "R. Ph" denotes relative pharmacological traits. "R.Ph" is determined by dividing the number of uses (U) by the total number of use reports in the whole study. "R.BS" indicates relative body systems treated. The "R.BS" value is obtained by dividing the number of body systems treated by a plant species by the total number of body systems studied.

### Fidelity Level (FL)

To determine the value of the species associated with medicines, the fidelity level was determined [16].

$$\mathrm{FL }(\mathrm{\%})\frac{{\text{Np}}}{{\text{N}}}\times 100$$

"Np" is the number of species in a particular category. "N" is used to accurately total consumption for specific species*.*

### Relative Popularity Level (RPL)

RPL is the ratio of the total number of informants for all diseases to the number of ailments healed by a specific plant species. Plant species with comparable FL may, however, have different therapeutic capacities. The relative popularity level (RPL) assumes a value between 0 and 1.0, with 0 denoting no ailments that a plant species treats and 1.0 denoting the entire popularity of a plant for significant ailments. The popularity index would be at its highest point (1.0) when all plant species were regularly employed to treat some serious illnesses; it would then decline toward zero as the relative popularity of the species veered away from the popular side. For species of popular plants, the RPL value is sensibly chosen to be unity (i.e., 1), but for species of unpopular plants, the RPL value is less than 1. It is determined whether a plant species is popular or unpopular based on its relative popularity level (RPL). Each plant's RPL value can be calculated based on its precise location on a graph [16].

### Rank Order Priority (ROP)

Plant species with different fidelity levels (FL) and Relative popularity level (RPL) values are properly ranked using a correction factor known as ROP. The ROP is made from the FL by multiplying the RPL and ROP values [16].

$${\text{ROP}}={\text{FL}}\times {\text{RPL}}$$

### Frequency index (FI)

To summarize the ethnobotanical data, a descriptive statistical method (percentage and/or frequency) was used. The Frequency Index (FI) was calculated using the following formula for the quantitative data analysis of the ethnomedicinal plants [17].

$${\text{FI}}=\frac{{\text{FC}}}{{\text{N}}}\times 100$$

Where “FC” is the number of traditional healers who mentioned the use of species and “N” is the total number of respondents.

### Cultural significance index (CSI)

The cultural significance index (CSI) was used to determine how well-aligned informant knowledge was with the use of reports for a particular species. The following formula was used to calculate [18].

$${\text{CSI}}=\sum ({\text{i}}\times {\text{e}}\times {\text{c}})\times {\text{CF}}$$

While "i" refers to the management of species that significantly affect the community, "e" indicates the informant's preference for one plant species over another for a specific purpose (value 2 for preferred species and value 1 for non-preferred species) and the letter "c" denotes the frequency of use of a plant species (a species that is cultivated, managed, or operated in any way receives a score of 2 and a score of 1 if the species is still free of any kind).

### Family use value (FUV)

Family use value (FUV) is a measure of the importance of plant species to a particular family. To determine the significance of plant families, the FUV is calculated by using this formula [18].

$${\text{FUV}}= \frac{\mathrm{\Sigma UVs}}{{\text{ns}}}$$

Where "ns" is the overall number of species within a family and "UVs" is the total usage value of all the species within that family.

### Preference Ranking (PR)

To discover which medicinal plant was best for each type of disease, a preference rating exercise was used. The medicinal plant that participants thought would be most effective in curing the stated diseases would be given the highest value in this activity. While the one that they thought would be the least successful would be given the lowest value. The scores for each species were added to determine the rank. This made it possible to identify the plant that the locals use to cure the conditions that are frequently reported [18].

### Jaccard index

This index is used to compare study data to other ethnobotanical studies undertaken in different countries around the world, as well as among indigenous groups in the examined locations. The formula for calculating the JI index [18]

$${\text{JI}}=\frac{\mathrm{c x }100}{({\text{a}}+{\text{b}})-{\text{c}}}$$

where “a” is the recorded number of species of the study area “A,” “b” is the documented number of species of the area “B” and “c” is the common number of species in both areas’ “A” and “B.”

## Results and discussion

Across diverse areas within Dunyapur, situated in District Lodhran, Pakistan, inhabitants employ varied remedies for a spectrum of illnesses. The present study emphasizes 41 plants spanning 28 families. Extensive field visits were conducted across multiple study sites to accumulate data on ethnomedicinal knowledge.

### Socio-demographic features of informants

In the course of fieldwork aimed at gathering essential ethnomedicinal information regarding medicinal plants, a total of 496 informants were interviewed regarding 41 plants, with 59% being men and 41% women. The predominant data collection occurred among adults aged 51 to 60. The documentation of indigenous traditional knowledge was facilitated through the utilization of a questionnaire and interview methodology, actively involving knowledgeable individuals such as farmers, Pansars, herbalists (Hakims), and other residents including housewives, LHVs, teachers, and shopkeepers as outlined in Table 1. The criteria for informant selection prioritized individuals with diverse expertise, with a majority consisting of farmers (35%), Pansars (12%), and herbalists (Hakims) (9%). The higher representation of these groups could be attributed to their significant roles in traditional knowledge about medicinal plants. Farmers often possess valuable insights into local flora due to their close interaction with the environment. Pansars, known for their expertise in traditional medicine and herbalists contribute specialized knowledge making them key informants. This distribution aligns with existing literature highlighting the prominence of these groups in ethnobotanical studies [19].

**Table 1:** Socio-demographic characteristics of informants

Variable	Demographic categories	Numbers	Percentage
**Gender**	Male	293	59
	Female	203	41
**Occupation**	Farmer	172	35
	Herbalist	47	9
	Pansar	57	12
	Other	220	44
**Age Groups**	31-40 year	45	9
	41-50 year	168	34
	51-60 year	198	40
	≥60 year	82	16
**Education**	Illiterate	141	28
	Primary	97	20
	Middle	86	18
	Matric	107	21
	Above Matriculation	65	13

### Growth habits of wild ethnomedicinal flora

**Fig. 4:**
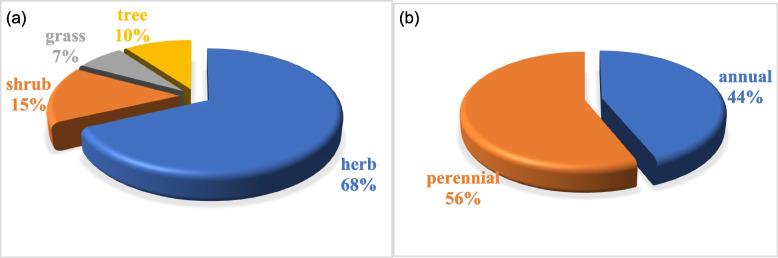
Growth habits of wild ethnomedicinal flora (**a**) Life span (**b**) life form

The current research documented a total of forty-one plant species, with herbs comprising the majority (68%), followed by shrubs (15%), trees (10%), and grasses (4%). The predominant life span preference in the study area was perennial (56%), with annual species accounting for the remaining 44%. All these species were reported to possess specific medicinal uses, as illustrated in Fig. 4a and b. Notably, among the fifty-one therapeutic plants identified, herbs constituted the majority, encompassing 57% of the total. Trees followed, with fifteen species, along with seven species of shrubs, most of which were perennial, although some were annual and biennial species [20]. This aligns with findings from a survey in the Shigar valley, Northern Pakistan, where herbs were also the most frequently utilized (51%), followed by shrubs (40%), trees (7%), and shrublets (2.85%) [21]. In our study, herbs were the most commonly documented growth habit, followed by shrubs and trees, respectively. The dominance of herbs in the current research, comprising 68% of the documented plant species, may be attributed to several factors consistent with findings in existing literature. Herbs often exhibit faster growth rates, adaptability to various ecological conditions, and shorter life cycles, making them more accessible for local communities in need of immediate therapeutic solutions. Additionally, herbs are commonly found in diverse ecosystems and are easily cultivated, enhancing their prevalence in traditional medicinal practices. The comparatively lower representation of shrubs, trees, and grasses (15%, 10%, and 4% respectively) could stem from their longer life cycles, slower growth rates, and specific habitat requirements, limiting their abundance in the local landscape. This aligns with findings in the literature that highlight the preference for herbs in ethnomedicinal uses due to their versatility, ease of availability, and rapid regrowth capacities, contributing to their prominence in traditional healing practices.

### Method of preparation

**Fig. 5:**
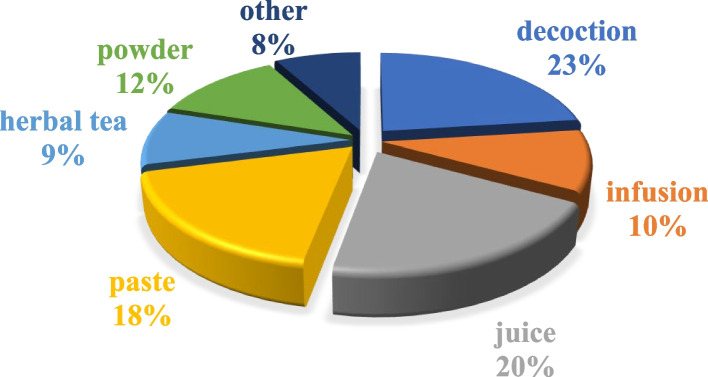
Percentage of different modes of preparation to cure diseases

The preparation of remedies from the 41 documented plant species was categorized based on their applications in medicinal formulations. The study revealed that decoction (23%) emerged as the predominantly utilized preparation technique among the locals, followed by juice (20%), paste (18%), powder (12%), infusion (10%), herbal tea (9%), and other methods (8%), encompassing tincture, ointment, lotion, steam, burning smoke and maceration (Fig. 5). Comparative analysis with existing studies corroborated these findings, highlighting the prevalence of decoction as the most utilized method in herbal remedy preparation. Notably, decoction was identified as the most preferred technique (17%) in a prior study, followed by paste (15%), powder (13%), extract (12%), and juice (13%) [22]. Another ethnobotanical study underscored the versatility of local remedies, indicating that decoction (71.4%), extract (66.7%), infusion, paste (38.1%), and powder (33.3%) were the primary modes of preparation, with juice and ash being less commonly employed (14.3%) [23]. The dominance of decoction, juice, and paste in medicinal preparations may be attributed to their efficacy, simplicity, and cultural preferences as suggested by existing literature. The versatility and ease of application of these methods contribute to their widespread use in traditional medicine.

### Quantitative ethnobotanical data analysis

The quantitative ethnobotanical analysis includes the calculation of some indices which are explained below.

### Plant part value (PPV)

**Fig. 6:**
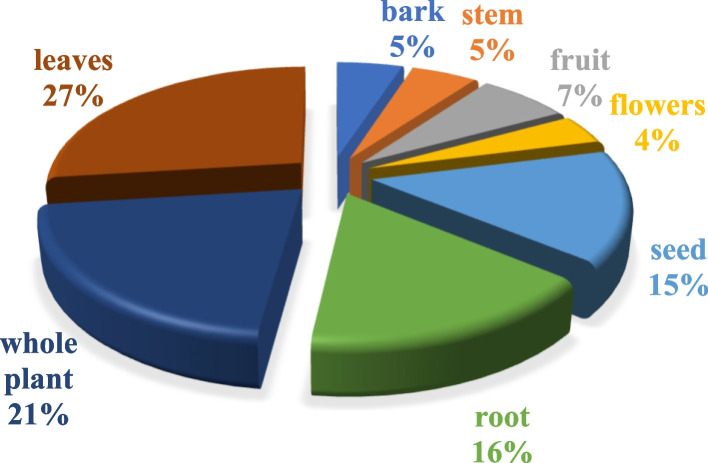
Percentage of various parts of the plant in ethnomedicinal application

In the research area, community members employed various plant components, either in combination or independently, to address human ailments. The most frequently utilized part in herbal remedies was leaves (27%), succeeded by whole plants (21%) and roots (16%) (Fig. 6). The prevalence of these particular plant parts in traditional medicine may stem from their practicality and accessibility. Leaves, for instance, are often easily harvested without causing harm to the plant, ensuring sustainability in herbal preparations. Comparative analysis with other studies revealed a similar pattern, where leaves were the most popular plant part in herbal remedies, accounting for 30.2% of usage. Locals' preference for leaves could be attributed to their ease of collection and widespread availability. Moreover, various studies consistently highlight leaves as the primary plant part employed in traditional recipes, emphasizing their bioactive compounds such as alkaloids, flavonoids, and essential oils, which contribute to potential health benefits. The recurring mention of leaves across different studies underscores their significant role in herbal medicine [24].

### Family use value (FUV)

**Fig. 7:**
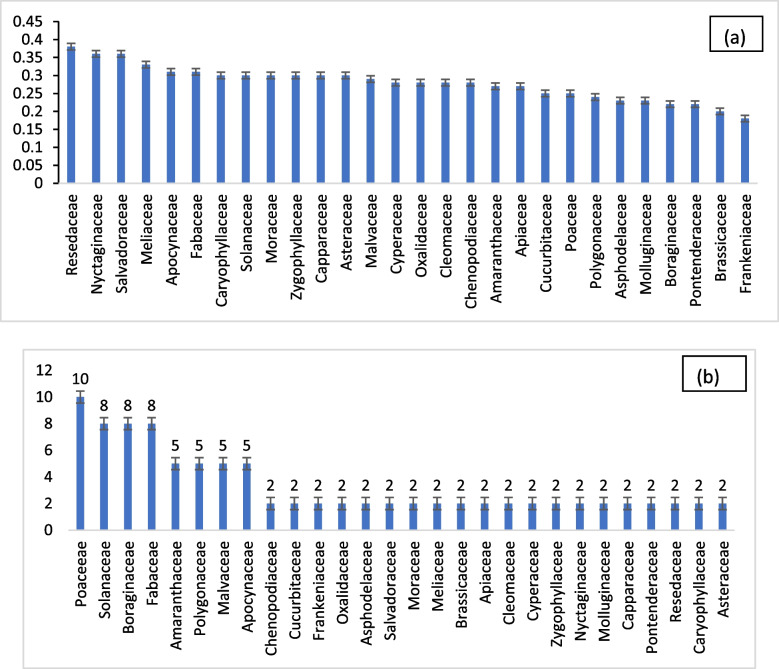
**a** Family Use Value (FUV). **b** Percentage of Family Importance Value (FIV)

Resedaceae attained the highest use value score of 0.38 in our study, underscoring its significant role in traditional medicinal practices (Fig. 7a). Nyctaginaceae and Salvadoraceae followed closely with scores of 0.36, while Meliaceae and Apocynaceae garnered scores of 0.33. Fabaceae registered a score of 0.31, with Caryophyllaceae, Zygophyllaceae, Solanaceae, Moraceae, Capparaceae, and Asteraceae each having a score of 0.3. Malvaceae scored 0.29, and Cyperaceae, Oxalidaceae, Cleomaceae, and Chenopodiaceae each received a score of 0.28. Amaranthaceae and Apiaceae shared a score of 0.27, while Cucurbitaceae and Poaceae scored 0.25. Polygonaceae obtained a score of 0.24, while Asphodelaceae and Molluginaceae both secured a score of 0.23. Boraginaceae and Pontederaceae earned a score of 0.22 each, and Brassicaceae obtained a score of 0.2, while Frankeniaceae had a score of 0.18. The families with the highest FUVs, denoting cultural significance, were Aloaceae, Acoraceae, and Piperaceae scoring 0.86, 0.80, and 0.69 respectively, in contrast to our findings (0.65). It's noteworthy that the highest FUV in our study was reported for Resedaceae (0.38), followed by Nyctaginaceae and Salvadoraceae (0.36 each). The comparison underscores the variability in family use values across regions and ecosystems, as observed in Colombia, where plant families like Asteraceae and Apiaceae did not consistently exhibit maximum FUVs, and families with only one plant species, such as Aristolochiaceae, demonstrated significant values. In our study, Caryophyllaceae led with a high FUV of 0.163, followed by Lamiaceae (FUV = 0.106), Apiaceae (FUV = 0.099), Rhamnaceae (FUV = 0.084), Asteraceae (FUV = 0.083), Poaceae (FUV = 0.074), Rutaceae (FUV = 0.044), Thymelaeaceae and Urticaceae (FUV = 0.036 each), Cucurbitaceae (FUV = 0.034), and Ericaceae (FUV = 0.024). Families with a usage value less than 0.024 were deemed less significant in terms of cultural use in this context. The variation in family use values highlights the importance of considering local contexts and ecological factors in understanding the cultural significance of plant families across different regions [25].

The high FUV of Nyctaginaceae and Salvadoraceae (both with a score of 0.36) in our study could be attributed to several factors. First, these plant families may have a rich diversity of species with significant traditional uses in the local context. The presence of plants from the Nyctaginaceae and Salvadoraceae families that are frequently utilized for medicinal, cultural, or economic purposes might contribute to their elevated FUV. Additionally, these families may encompass species with versatile applications, addressing various human needs, thus increasing their cultural significance. Comparisons with available literature would be beneficial to discern whether the findings align with patterns observed in other regions and ecosystems. On the other hand, Cucurbitaceae (FUV = 0.034) and Ericaceae (FUV = 0.024) having comparatively lower FUVs could be due to factors such as limited cultural uses or a lower diversity of species with significant traditional importance in the study area. The reduced FUV might indicate that the plants from these families are less frequently employed or have a narrower range of applications in the local context. The literature should be consulted to explore whether the lower FUV for Cucurbitaceae and Ericaceae aligns with patterns observed in other regions, shedding light on the cultural and ecological factors influencing the significance of these plant families.

### Family Importance Value (FIV)

Among the 41 plants studied, a total of 28 different families were identified. Poaceae emerged as the largest family with four species, constituting 11% of the total, followed by Boraginaceae, Fabaceae, and Solanaceae, each comprising 9% with three species each. Amaranthaceae, Polygonaceae, Apocynaceae, and Malvaceae accounted for 5% with two species each. Cyperaceae, Chenopodiaceae, Cucurbitaceae, Frankeniaceae, Oxalidaceae, Asphodelaceae, Salvadoraceae, Moraceae, Meliaceae, Brassicaceae, Apiaceae, Cleomaceae, Zygophyllaceae, Nyctaginaceae, Molluginaceae, Capparaceae, Pontenderaceae, Resedaceae, Caryophyllaceae, and Asteraceae comprised the lowest percentage at 2%, each with one species (Fig. 7b). In Toba Tek Singh District, 16 different plant families were represented, with Fabaceae leading with five species, followed by Asteraceae and Amaranthaceae tying for second place with four species each. Poaceae and Euphorbiaceae had two species each. In Haroon Abad, district Bahawalnagar, the natural flora consisted of 81 species across 21 families, with Poaceae dominating as the largest family with 15 species, followed by Euphorbiaceae (8 species), Asteraceae (7 species), and Amaranthaceae (7 species). The distribution of species across families varied in different regions, reflecting the ecological diversity and local conditions influencing plant composition [26].

The high FIV of Poaceae, Boraginaceae, Fabaceae, and Solanaceae in our study, with each family having three species, may be attributed to several factors. First, these families could exhibit a significant presence in the study area, with multiple species playing crucial roles in local ecosystems, traditional practices, or cultural significance. The ecological abundance and versatility of these families might contribute to their elevated FIV. Additionally, these families may encompass plant species with diverse uses, addressing various human needs such as medicinal, cultural, or economic purposes. The families' collective importance could be a result of the combined utility of their constituent species in the daily lives of the local community, making them stand out in terms of ecological and cultural significance. Comparisons with available literature and ecological studies specific to the region could provide further insights into the reasons behind the high FIV of Poaceae, Boraginaceae, Fabaceae, and Solanaceae in the study area. Understanding the ecological, cultural, and economic roles of these families in the local context would shed light on their importance in the studied ecosystem.

### Popular therapeutic use value (POPUT)

In our study, various plant species exhibited distinct Medicinal Use Values, reflecting how respondents utilize specific plants for particular diseases. In the current research, these values ranged from 0.6 to 0.004. The highest value was attributed to analgesic effects (0.6), followed by jaundice (0.48), ulcer treatment (0.3), fever management (0.07), and equal values for diarrhea and skin diseases (0.006 each). Inflammation, cough, liver diseases, cancer, and kidney diseases shared a value of 0.04, while stomach-related ailments, asthma, piles, gargles, eye diseases, rheumatism, snake bites, hepatitis, and constipation were assigned values of 0.03 each. Wound healing received a value of 0.02, while diuretics, gonorrhea, laxatives, cardiac diseases, and blood purification were associated with 0.01 each. Anti-microbial properties and pain management were linked to values of 0.006, while the lowest values were recorded for cholesterol and diabetes (0.004 each). Respondents ranked these values from the highest to the lowest based on their perceptions (Table 2). Comparatively, a Turkish study presented diverse POPUT values, with shortness of breath at 0.11, abdominal discomfort at 0.10, wound treatment at 0.08, and stomach issues at 0.07, among other examples. The current investigation provides unique insights, highlighting analgesic properties with the highest value (0.6), while cholesterol and diabetes received the lowest values (0.004 each).

**Table 2:** Informant Consensus Factor (ICF) Popular and Therapeutic Use Value (POPUT) of different ailments

Disease Name	ICF	POPUT
Analgesic	0.92	0.6
Anti-microbial	1.00	0.006
Asthma	0.73	0.03
Blood purifier	1.00	0.01
Cancer	0.72	0.04
Cardiac diseases	1.00	0.01
Cholesterol	1.00	0.004
Constipation	0.78	0.03
Cough	0.73	0.04
Diabetes	0.60	0.006
Diarrhea	0.75	0.06
Diuretic	0.76	0.01
Eye diseases	0.81	0.03
Fever	0.77	0.07
Gargles	1.00	0.03
Gonorrhea	1.00	0.001
Hepatitis	0.83	0.03
Inflammation	0.65	0.05
Jaundice	0.76	0.48
Kidney diseases	0.87	0.04
Laxative	0.83	0.01
Liver diseases	0.75	0.04
Pain	0.83	0.006
Piles	0.80	0.03
Rheumatism	0.75	0.03
Skin diseases	0.72	0.06
Snake bite	1.00	0.03
Stomach	0.83	0.03
Ulcer	0.69	0.3
Wound healing	0.72	0.02

The observed high POPUT values for analgesia, jaundice, ulcer, fever, diarrhea, and skin diseases (ranging from 0.6 to 0.006) in our study could be influenced by the prevalence and perceived effectiveness of traditional remedies for these particular health concerns in the local community. Analgesic properties are often sought after for pain relief, which might contribute to its prominent ranking. Similarly, jaundice, ulcers, fever, diarrhea, and skin diseases are commonly encountered health issues, and traditional medicinal practices often focus on addressing these ailments. The local abundance of plants with known efficacy against these specific health concerns could also contribute to their higher POPUT values. However, variations in the prevalence of certain diseases and the cultural significance attached to them could explain the differences in POPUT values. Comparisons with available literature and ethnobotanical studies specific to the region can provide insights into the cultural and ecological factors influencing the popularity of certain therapeutic uses over others [27].

### Informant Consensus Factor (ICF)

The ICF serves as a metric indicating the degree of association between specific plant species and their use in treating particular ailments. In our survey, ICF values ranged from 0.6 to 1, with the highest values assigned to cholesterol, cardiac diseases, antimicrobial properties, snake bites, blood purification, gonorrhea, and gargles (1 for each). Subsequent high values were recorded for analgesic (0.92), kidney-related issues (0.87), laxative, pain, hepatitis, and stomach ailments (0.83 for each), followed by eye diseases (0.81), piles (0.8), constipation (0.78), fever (0.77), diuretic, jaundice (0.76), rheumatism, liver conditions, and diarrhea (0.75), cough, asthma (0.73), cancer, skin diseases, and wound healing (0.72), ulcer (0.69), inflammation (0.65), and diabetes (0.60) (Table 2). In a parallel ethnobotanical survey in Tehsil Gojra, District Toba Tek Singh, high ICF values were documented for mental disorders and epilepsy (1), possibly attributed to the infrequency of these diseases in the region, leading to a focused use of specific plant species. This contrasts with our findings, where maximum ICF values were assigned to cholesterol, cardiac diseases, antimicrobial properties, blood purification, snake bites, gonorrhea, and gargles. Another survey indicated ICF values ranging from 0.66 to 0.93 for constipation and respiratory problems, with an average ICF value of 0.87 for plant utilization in treating various ailments among informants [28]. The highest ICF values, reaching 1, for cholesterol, cardiac diseases, antimicrobials, snake bites, blood purification, gonorrhea, and gargles in our study could be attributed to the specific cultural and medicinal significance assigned to plants addressing these health concerns in the local community. Certain diseases, such as cardiac issues and infections, might be prevalent or culturally prioritized, leading to a concentrated use of plants believed to be effective against them. Analgesic (0.92), kidney-related issues (0.87), laxative, pain, hepatitis, stomach ailments (0.83 for each), and eye diseases (0.81) also received relatively high ICF values, suggesting their perceived importance in the traditional pharmacopeia. The variation in ICF values may be influenced by factors such as disease prevalence, cultural beliefs, and the perceived efficacy of traditional remedies.

### Preference ranking

For skin disorders, the most used plant was *M. azedarach* (25) which was followed by *P. harmala* (24), *C. jwarancusa* (22), *E. crassipes* (20), *S. oleoides* (19), *T. resupinatum* (18) and *H. curassavicum* (14). As a diuretic, the most reported plant was *A. tenuifloius* (23), followed by *D. muricata* (21), *C. conglomeratus* (20), *B. diffusa* (19) and *C. album* (14). *S. oleoides* (24) was considered the most effective against cough and was ranked at the top among other species. It was followed by *C. spinosa* (22), *C. gigantea* (20), *H. europaeum* (19), *C. jwarancusa* (18), *T. resupinatum* (16) and *M. sylvestris* (14). The most preferred plant for inflammation was *P. aculeata* (20) which was followed by *C. jwarancusa* (19), *C. brachycarpa* (17), *G. lotoides* (16), *M. albus* (15), *D. metel* (14) and *A. indicum* (12) which was lowest value (Table 3). According to the findings of previous research which ranked the preferences of seven medicinal plants used to treat high blood pressure, *Verbascum sinaiticum* received the highest score, earning a score of 74 [29]. In another study, stomach discomfort was treated with 21% of the therapeutic plants found in the research region. *Rumex nepalensis* was the most widely used treatment for stomach problems and it was followed in popularity by *Ruta chalepensis*, *Clausena anisata* and *Zingiber officinale*. Following a ranking of six plants that can treat malaria, *Allium sativum*, the most effective medicinal herb, took the top position [30]. The high PR values for *M. azedarach* (25), *P. harmala* (24), *C. jwarancusa* (22), *E. crassipes* (20) and *S. oleoides* (19) in the treatment of skin-related issues suggest their widespread acceptance and effectiveness in local community. Various factors contribute to these high rankings including plants' accessibility, ease of use, cultural significance and perhaps most importantly perceived efficacy based on traditional knowledge. Local communities often prioritize plants with a history of successful use passing down knowledge from generation to generation. Additionally, the unique biochemical composition of these plants may play a role in their effectiveness for ailments further enhancing their preference. It is crucial to consider the collective wisdom embedded in local practices when interpreting these preference rankings as they reflect the community's trust and reliance on specific plant species for addressing skin-related concerns.

**Table 3:** Preference Ranking (PR)

Plant name	Diseases	Respondents (R1-R10)											
		**R1**	**R2**	**R3**	**R4**	**R5**	**R6**	**R7**	**R8**	**R9**	**R10**	**Score**	**Rank**
*Melia azedarach* L.	Skin	3	2	3	2	3	2	3	3	1	3	25	1^st^
*Peganum harmala* L.		3	2	3	3	2	2	2	3	2	2	24	2^nd^
*Cymbopogon jwarancusa (Jones ex Roxb.) Schult.*		3	2	2	2	3	1	3	3	3	0	22	3^rd^
*Eichhornia crassipes (Mart.) Solms*		3	1	3	1	3	1	3	1	2	2	20	4^th^
*Salvadora oleoides* Decne.		0	2	2	2	2	2	2	2	3	2	19	5^th^
*Trifolium resupinatum* L*.*		1	2	2	3	1	3	2	1	1	2	18	6^th^
*Heliotropium curassavicum* L.		1	2	2	1	2	1	1	1	3	0	14	7^th^
*Asphodelus tenuifloius* Cav.	Diuretic	1	2	3	2	2	2	3	3	2	3	23	1st
*Digera muricata Mart.*		3	2	2	2	3	3	1	1	3	1	21	2^nd^
*Cyperus conglomeratus* Rottb.		3	1	1	2	3	2	1	2	2	3	20	3^rd^
*Boerhavia diffusa* L.		2	1	3	3	1	1	3	1	2	2	19	4^th^
*Chenopodium album* L.		3	3	3	1	1	3	0	0	0	0	14	5^th^
*Salvadora oleoides* Decne.	Cough	2	3	2	3	3	2	2	3	2	2	24	1st
*Capparis spinosa* L*.*		2	3	2	1	3	2	1	3	2	3	22	2^nd^
*Calotropis gigantea (L.) Dryand.*		3	2	3	2	3	3	1	1	0	2	20	3^rd^
*Heliotropium europaeum* L*.*		3	2	1	1	1	3	2	2	2	2	19	4^th^
*Cymbopogon jwarancusa (Jones ex Roxb.) Schult.*		1	2	1	1	2	3	3	3	1	1	18	5^th^
*Trifolium resupinatum* L.		3	1	1	2	3	2	2	1	1	0	16	6^th^
*Malva sylvestris* L*.*		1	2	1	2	1	1	1	2	3	1	15	7^th^
*Glinus lotoides* L.	Wound healing	3	2	3	1	2	3	3	1	1	3	22	1^st^
*Aeluropus lagopoides (L.) Trin. ex Thwaites*		3	1	2	3	2	3	2	2	3	1	22	2^nd^
*Nicotiana plumbaginifolia* Viv*.*		1	1	2	2	2	3	2	3	2	3	21	3^rd^
*Heliotropium europaeum* L.		2	1	1	2	2	2	3	1	2	2	18	4^th^
*Heliotropium curassavicum* L*.*		2	2	1	2	1	2	1	2	2	3	18	5^th^
*Malva sylvestris* L.		2	1	2	1	2	1	2	2	1	3	17	6^th^
*Parkinsonia aculeata* L*.*	Inflammation	2	3	2	3	2	2	2	3	1	2	22	1^st^
*Cymbopogon jwarancusa (Jones ex Roxb.) Schult.*		2	3	1	2	2	1	3	2	3	2	21	2^nd^
*Cleome brachycarpa M.Vahl ex Triana & Planchon*		1	2	2	1	1	2	2	3	3	1	18	3^rd^
*Glinus lotoides* L.		3	1	2	1	1	2	1	2	3	1	17	4^th^
*Melilotus albus* Desr.		2	1	2	1	1	3	2	3	0	1	16	5^th^
*Datura metel* L.		1	1	2	3	1	3	1	2	0	0	14	6^th^
*Abutilon indicum* L. Sweet		2	1	2	1	2	1	2	1	0	0	12	7^th^

^*^Success rate of this plant for treatment 3= high, 2= average, 1=low R1-R8= Respondents

### Relative frequency citation (RFC)

RFC is used to calculate the significance of a specific plant species in a given community or culture in scientific research. In the current research, its value ranged from 0.016 to 0.032. The highest value was reported for *C. melo* (0.032) which was followed by *P. glabra, P. barbata, L. pyrotechnica* (0.03 for each), *A. indicum, O. corniculata, S. virginianum, C. conglomeratus, C. jwarancusa, C. brachycarpa, C. album, H. europaeum* (0.028 for each). The lowest values were calculated for *D. bipinnata, A. graveolens, T. resupinatum, H. curassavicum, B. diffusa, M. albus* (0.022 for each), *F. benghalensis, C. gigantea, M. africana, P. aculeata, P. harmala, L. perenne, M. sylvestris* (0.02 for each), *E. crassipes* (0.018) and lowest (0.016) was reported for *O. linifolia, A. lagopoides* (Table 4). *Skimmia laureola* had the maximum RFC value during research in Pakistan's Hindukush Range of District Swat at 0.321 followed by *Juglans regia, Olea europaea* and *Papaver somniferum* (0.294 each*)* [30]. In Azad Jammu and Kashmir RFC values varied from 0.93 to 0.04. For *Berberis lyceum* the highest RFC value of 0.81was recorded. Other plants with notable RFC values included *Ajuga bracteosa, Prunella vulgaris, Adiantum capillus-veneris, Desmodium polycarpum, Punica granatum* and *Jasminum mesnyi*. The maximum RFC values were found for *Ficus palmata* (0.42), *Cicer arietinum* (0.36) and *Chenopodium album* (0.36) whilst the lowest RFC values were found in *Rosa damascena* (0.13) and 0.016 for *Aeluropus lagopoides* in our study. According to most interviewees, it suggests that these species are the most known and often used herbs in the research area [31]. The high RFC values reported for specific plants in the current research such as *C. melo* (0.032), *P. glabra, P. barbata, L. pyrotechnica* (0.03 for each), *Abutilon indicum, Oxalis corniculata, Solanum virginianum, Cyperus conglomeratus, C. jwarancusa, C. brachycarpa, C. album* and* H. europaeum* (0.028 for each) suggest their frequent citation and cultural significance within the community. The RFC value is calculated based on how often a plant is mentioned by respondents about its medicinal uses. These plants may have higher RFC values due to their widespread recognition, traditional importance and effectiveness in treating various ailments within the local context.

**Table 4:** Ethnobotanical plant traits used by indigenous people in the study area

BN	LN	Family	LF	PU	Rec.	App.	Uses	UV	FL	RI	RFC	FI	RPL	ROP	CSI	Previously Reported
*Abutilon indicum* (L.) Sweet 362/Botany	Kanghi booti	Malvaceae	Perennial shrub	Whole plant	Paste, Infusion	Massage, Oral	Piles, Inflammation, cancer, diarrhea	0.28	31	3.56	0.007	0.77	0.20	18.75	3.5	
*Aeluropus lagopoides *(L.) Trin. ex Thwaites 397/Botany	Pooji Chabbar	Poaceae	Perennial grass	Whole plant	Herbal tea, paste	Oral, Topical	Diabetes, wound healing	0.25	62	1.8	0.004	0.44	0.33	10.61	0.5	
*Anethum graveolens* L. 382/Botany	Soay	Apiaceae	Annual herb	Seeds, leaves	Decoction, herbal tea	Oral	Eye diseases, stomach, ulcer	0.33	44	1.83	0.004	0.49	0.30	14.52	0.56	
*Asphodelus tenuifloius* Cav. 375/Botany	Piazzi	Asphodelaceae	Annual herb	Seed, root, stem, leaves	Juice, herbal tea and decoctions	Oral, topical	Wound healing, diuretic, piles	0.27	54	2.68	0.006	0.6	0.27	14.72	2.75	
*Boerhavia diffusa* L. 389/Botany	It-sit	Nyctaginaceae	Perennial herb	Root, leaves	Juice, powder	Oral, Topical	Jaundice, eye diseases, diuretic *Diabetes	0.4	40	3.47	0.005	0.55	0.30	16	2.35	
*Calotropis gigantea *(L.) Dryand. 380/Botany	Bara_ak	Apocynaceae	Perennial shrub	Whole plant	Juice, Fresh paste	Oral, Topical massage	Cough, asthma, *fever	0.33	50	3.5	0.006	0.66	0.30	16.5	1.5	
*Capparis spinosa* L. 392/Botany	Kuber	Capparaceae	Perennial shrub	Fruit, leaves, root, bark	Herbal tea, powder paste	Oral, Topical, Massage	Diabetes, cough, asthma, pain	0.3	30	3.53	0.007	0.71	0.21	13.8	1.73	
*Carthamus oxyacantha *M.Bieb. 398/Botany	Pohli	Asteraceae	Annual herb	Whole plant	Juice, decoction	Oral, Massage	Ulcer, jaundice, skin diseases, *diarrhea	0.33	42	3.5	0.006	0.66	0.22	0.13	3.2	
*Chenopodium album* L. 369/Botany	Bathu	Chenopodiaceae	Annual herb	Seeds, leaves, shoots	Decoction, Herbal tea powder	Oral,Massage	Stomachache, laxative, diuretic, hepatitis	0.25	57	2.71	0.006	0.66	0.25	14.25	1.6	
*Cleome brachycarpa *M.Vahl ex Triana & Planchon 368/Botany	Kastoori	Cleomaceae	Perennial herb	Whole plant	Decoction, paste	Oral, Topical, Massage	Skin diseases, anti-microbial, rheumatism, inflammation	0.27	38	2.79	0.008	0.82	0.28	10.26	3.75	
*Cucumis melo* L. 361/Botany	Chibbar	Cucurbitaceae	Annual herb	Whole plant	Herbal tea, infusion, decoction, paste	Oral, Topical	Cholesterol, constipation, kidney, diuretic	0.36	70	2.68	0.006	0.6	0.23	7.6	1.375	
*Cymbopogon jwarancusa *(Jones ex Roxb.) Schult. 367/Botany	Khawi	Poaceae	Perennial herb	Whole plant	Paste, decoction	Oral, Topical, Massage	*Gargles, skin diseases, cough, inflammation	0.23	37	2.73	0.007	0.71	0.25	16.15	3.058824	
*Cyperus conglomeratus *Rottb. 400/Botany	Deela	Cyperaceae	Perennial herb	Root, leaves, fruit	Decoction, paste	Topical, Massage	Stomach, diuretic, constipation	0.28	36	3.56	0.007	0.77	0.30	10.29	3.73	
*Datura metel *L. 364/Botany	Datura	Solanaceae	Annual shrub	Whole plant	Decoction, infusion, paste	Oral	Skin diseases, asthma, cough inflammation	0.4	40	3.47	0.005	0.55	0.30	16	2.35	
*Desmostachya bipinnata *(L.) Stapf 378/Botany	Dab	Poaceae	Perennial grass	Whole plant	Juice, paste	Oral	Inflammation, fever, asthma	0.21	43	2.76	0.007	0.77	0.36	9.21	1.86	
*Digera muricata *Mart. 370/Botany	Taandla	Amaranthaceae	Annual, herb	Whole plant	Paste, decoction, herbal tea, juice	Oral, Topical	Constipation, diuretic, liver, diabetes	0.28	43	3.56	0.007	0.77	0.30	12.29	3.73	
*Eichhornia crassipes *(Mart.) Solms 393/Botany	Jal kudi	Pontenderaceae	Perennial herb	Stem, Leaves, root	Juice, infusion	Oral	*Cancer, skin diseases	0.21	42	2.76	0.007	0.77	0.36	8.82	3.5	
*Ficus benghalensis* L. 377/Botany	Bohar	Moraceae	Perennial tree	Stem, root, leaves, flowers	Powder, juice	Oral	*Wound healing, gonorrhea, diarrhea	0.27	30	2.65	0.005	0.55	0.33	8.1	1.25	
*Frankenia pulverulenta* L. 372/Botany	Khareya	Frankeniaceae	Annual herb	Flowers, stem, leaves	Decoction, paste	Oral, Massage	Skin diseases, analgesic	0.27	45	2.65	0.006	0.6	0.27	12.2	2.58	
*Glinus lotoides* L. 390/Botany	Gandi booti	Molluginaceae	Annual herb	Whole plant	Juice, decoction, paste	Oral, Topical	Inflammation, ulcer, wound healing	0.33	40	2.79	0.004	0.49	0.28	13.3	1.05	
*Heliotropium crispum* Desf. 374/Botany	Kaali lani	Boraginaceae	Perennial herb	whole plant	Decoction, powder	Oral, Topical	Usage in ulcer and kidney diseases	0.23	53	2.73	0.007	0.71	0.30	12.3	3.25	
*Heliotropium curassavicum* L. 388/Botany	Lani patta	Boraginaceae	Perennial herb	Root, leaves, flowers	Infusion, paste	Oral, Topical	Used for wound healing, skin and hepatitis	0.17	83	1.91	0.006	0.66	0.28	14.16	3	
*Heliotropium europaeum* L. 373/Botany	Haathi sundi	Boraginaceae	Annual herb	Whole plant	Decoction, infusion	Oral, Topical	Skin, wound healing, cough	0.27	45	2.68	0.006	0.6	0.23	12.27	2.75	
*Leptadenia pyrotechnica* (Forssk.) Decne. 385/Botany	Khipp	Apocynaceae	perennial shrub	Whole plant	Paste, decoction	Oral, Massage	Cancer, fever, jaundice, constipation, diabetes	0.4	30	2.71	0.005	0.66	0.27	12	2.35	
*Lolium perenne* L. 391/Botany		Poaceae	Perennial grass	whole plant	Infusion, paste	Oral, Massage	*Diarrhea, cancer	0.3	50	2.65	0.005	0.55	0.33	15	2.67	
*Malcolmia africana *(L.) W.T.Aiton 381/Botany	Jangle surmee	Brassicaceae	Annual herb	Flowers, leaves, seed	Powder, paste	Oral, Topical	*Used in diabetes and cardiac diseases	0.2	80	1.86	0.005	0.55	0.33	16	1.25	
*Malva sylvestris* L. 394/Botany	Khubazi	Malvaceae	Perennial herb	Leaves, root	Decoction, paste	Oral	Wound healing, cough, ulcer	0.2	80	1.86	0.005	0.55	0.28	16	2.5	
*Melia azedarach* L. 379/Botany	Bakain	Meliaceae	Perennial tree	Seed, leaves, bark	Decoction, juice	Oral	Ulcer, blood purify, skin diseases	0.2	50	1.89	0.005	0.55	0.17	10	2.35	
*Melilotus albus* Desr. 399/Botany	Jangle sainjee	Fabaceae	Annual herb	Seeds, Leaves	Infusion, juice	Oral	*Laxative, inflammation, eye diseases	0.22	66	3.5	0.006	0.66	0.28	14.52	0.56	
*Nicotiana plumbaginifolia *Viv. 401/Botany	Jangle tambaku	Solanaceae	Annual herb	Whole plant	Juice, paste	Oral, Topical	Inflammation, rheumatism, snake bite, wound healing	0.3	40	3.48	0.005	0.55	0.27	12	2.5	
*Oligomeris linifolia *J.F.Macbr. 395/Botany	Shootk	Resedaceae	Annual herb	Leaves, fruit	Powder, decoction	Oral	*Fever, jaundice, diarrhea	0.33	33	3.5	0.006	0.66	0.38	10.98	3.00	
*Oxalis corniculata* L. 363/Botany.	Khati methi booti	Oxalidaceae	Perennial herb	Whole plant	Decoction, infusion	Oral	Diuretic, diabetes, stomach, fever	0.38	50	2.6	0.004	0.44	0.28	19	0.5	
*Parkinsonia aculeata* L. 383/Botany	Walaiti kikar	Fabaceae	Perennial tree	Whole plant	Decoction, juice	Oral	Diabetes, inflammation	0.3	40	2.65	0.005	0.55	0.30	12	2.67	
*Peganum harmala* L. 387/Botany	Harmal	Zygophyllaceae	Perennial herb	Seed, root, bark	Decoction, infusion, paste	Oral, Massage	*Diabetes, skin diseases, asthma	0.27	36	2.62	0.006	0.49	0.30	9.72	2.75	
*Persicaria barbata *(L.) Hara 384/Botany	Jor booti	Polygonaceae	Annual herb	Whole plant	Powder, infusion	Oral, Massage	Wound healing, rheumatism, ulcer, astringent	0.44	44	2.65	0.004	0.55	0.27	19.5	1.05	
*Persicaria glabra *(Willd.) M.Gómez 365/Botany	Booti, gandi booti	Polygonaceae	Annual herb	Whole plant	Decoction, infusion, paste	Oral, Topical	*Diuretic, pain, cancer	0.27	40	3.58	0.008	0.49	0.23	10.8	1.875	
*Salvadora oleoides* Decne. 376/Botany	Wan	Salvadoraceae	Perennial tree	whole plant	Decoction, paste, herbal tea	Oral, Topical	*Analgesic, skin diseases, cough, rheumatism,	0.21	50	3.58	0.007	0.77	0.20	10.5	1.125	
*Silene conoidea* L. 396/Botany	Chota takla	Caryophyllaceae	Annual herb	Leaves, bark, stem	Decoction, paste	Oral, Topical	Cancer, pain, diabetes, skin diseases	0.18	44	2.62	0.008	0.88	0.20	8.25	2.82	
*Solanum virginianum *L. 366/Botany	Kantakari	Solanaceae	Perennial herb	Whole plant	Juice, paste, infusion	Oral, Topical	Liver disorders, cough, asthma, fever	0.27	45	2.68	0.006	0.6	0.18	12.2	3.05	
*Suaeda vera *Forssk. ex J.F.Gmel. 371/Botany	Laani	Amaranthaceae	perennial shrub	Whole plant	Powder, herbal tea	Oral	*Kidney diseases, skin, eyes problems	0.23	53	2.73	0.007	0.71	0.28	12.38	3.25	
*Trifolium resupinatum *L. 386/Botany	Shatala	Fabaceae	Annual herb	Whole plant	Juice, herbal tea	Oral	*Usage in cancer, ulcer, skin disorders, cough	0.36	36	3.47	0.006	0.6	0.30	13.17	2.5	

*BN* Botanical name, *LN* Local name, *LF* Life form, *PU* Part used Rec. *Recipe* App. Mode of application, *UV* Use value, *RFC* Relative frequency citation, *RI* Relative importance, *CSI* Cultural significance index, *FI* Frequency index, *RPL* Relative popularity level, *ROP* Rank order priority, *FL* Fidelity level, * = novel use, (Ψ) = plant with similar use; (φ) = plant with dissimilar use; (  ) = plant not reported in previous study  1: (Nisar *et al.,* 2014); 2: (Anwer *et al.,* 2020); 3: (Ali *et al.,* 2023); 4: (Shah *et al.,* 2013); 5: (Umair *et al.,* 2017); 6: (Wariss *et al., *2014); 7: (Uzun and Koca, 2020); 8: (Behera *et al.,* 2021); 9: (Guo *et al.,* 2022); 10: (Nadaf *et al.,* 2023). Ingestion of *M. azedarach* seeds can lead to symptoms such as vomiting, diarrhea, abdominal pain. *A. indicum* may cause allergic reactions. Ingestion of *C. gigantea* contain may cause nausea, vomiting, abdominal pain and in extreme cases, it can be fatal. In some cases, *C. album* may cause allergic reactions

### Use value (UV) of plant species

In our study, the UV varied from 0.17 to 0.38. The maximum value was documented for *O. linifolia* (0.38) which was followed by *S. oleoides, T. resupinatum* and *B. diffusa* (0.36 for each). For *M. azedarach, L. pyrotechnica, N. plumbaginifolia* (0.33 for each), *D. metel, F. benghalensis, C. gigantea, P. aculeata, P. harmala, C. spinosa, M. sylvestris, S. conoidea, C. oxyacantha* (0.3 for each), *A. indicum, O. corniculata, S. virginianum, C. jwarancusa, C. brachycarpa, C. conglomeratus, C. album* (0.28 for each), *D. bipinnata, A. graveolens, P. barbata, H. curassavicum, M. albus* (0.27 for each) and *H. crispum* (0.17) which was the lowest value in Table 4. In Kotli, its value varied from 0.2 to 0.96. The maximum UV was calculated for *Cuscuta reflexa* (0.96) and the lowest was calculated for *Rhamnus triquetra* (0.2) [32]. *Achyranthes aspera* has a high UV value indicating that it is frequently used in Bahawalpur to treat pneumonia, bronchitis, colds and other respiratory illnesses. *Ficus religiosa* (0.68), *Hyssopus officinalis* (0.5) and *Onosma bracteatum* are the medicinal plants with the lowest use value (0.32). UV values in Waziristan varied from 0.02 to 1.00. *Thymus mongolicus* had the highest recorded use value (UV=1) followed by *Mentha longifolia, Foeniculum vulgare* and *Juglans regia* with UVs of 0.97 and 0.94 respectively. The lowest UV values were found for *Adiantum capilus-vaneri*s (0.02) [33]. The UV calculation is influenced by the frequency and diversity of plant use within a specific community or culture. In our study, certain plants exhibited higher UV values such as *O. linifolia, S. oleoides, T. resupinatum* and *B. diffusa* (all with 0.36 or 0.38) indicating their greater significance in the local traditional knowledge and practices. The higher UV values for these plants may be attributed to their widespread use, versatile applications or perceived efficacy in treating various ailments. *O. linifolia* attained the maximum UV (0.38) suggesting that it is prominently recognized and extensively utilized in community. Additionally, *M. azedarach, L. pyrotechnica, N. plumbaginifolia* (each with 0.33) and other plants with relatively high UV demonstrate their cultural importance and potential therapeutic value. Comparisons with available literature and cross-cultural analyses can provide insights into the contextual and cultural factors influencing prominence of specific plant species. The reasons for higher UVs can be multifaceted encompassing factors such as local traditions, historical practices, perceived efficacy and the availability of these plants in the region.

### Relative Importance (RI)

In the current study, its value varied from 4.14 to 9.85. The highest value was calculated for *L. pyrotechnica* (9.85). It was followed by *C. melo* (8.28), *P. barbata* (8.18), *A. indicum, O. corniculata, C. album, C. jwarancusa, C. brachycarpa, S. virginianum* (8.08 for each), *D. metel, D. muricata, C. spinosa, S. conoidea, C. oxyacantha*, (7.98 for each). For *M. azedarach, N. plumbaginifolia* (7.88 for each), *S. oleoides, T. resupinatum, B. diffusa* (7.78 for each), *P. glabra* (6.51), *C. conglomeratus, H. europaeum* (6.41), *G. lotoides* (6.31), *C. gigantea, F. benghalensis, M. sylvestris* (6 for each), *O. linifolia* (5.81), *H. crispum* (4.54), *F. pulverulenta* (4.44), *M. africana, L. perenne* (4.34 for each), *E. crassipes* (4.25) and 4.14 for *A. lagopoides* (Table 4). In previous investigation, the RI varied from 0.97 to 100. The highest (100%) RI for curing a specific condition was achieved by *Lawsonia inermis, Punica granatum, Rosa indica,* and *Vitis vinifera* [34]. An ethnobotanical survey in Azad Jammu Kashmir showed different values of relatively important species of the area. Utilizing the RI value for treating various ailments, the variety of a species is evaluated. While *Verbascum thapsus* (79) and *Azadirachta indica* (73) had maximum RI values, *Galium aparine* (96) and *Mimosa pudica* (91) had the maximum RI value [35]. The higher RI values could be attributed to several factors including the plant's effectiveness in treating prevalent health conditions in the surveyed area, the cultural significance attached to these plants and historical practices passed down through generations. These plants may have established themselves as go-to remedies for common ailments, leading to their higher perceived importance. Additionally, factors like ease of accessibility, abundance and familiarity with these plants within the community might contribute to their higher RI values. The reasons for elevated RI values for specific plants can vary based on the local context, cultural beliefs and practical experiences of the community members highlighting the importance of considering these factors in ethnobotanical studies.

### Fidelity Level (FL)

The highest FL was reported for *H. crispum* (83.33%) for curing kidney diseases, *M. africana* (80%) for cardiac diseases, *L. perenne* for cancer (80%), *E. crassipes* for skin diseases (77.78%), *D. bipinnata* for fever (63.63%), *A.* l*agopoides* (62.5) for wound healing, *F. pulverulenta* as analgesic (61.53%), *G. lotoides* (53.84%) for wound healing and *C. conglomeratus* (50%) as diuretic (Table 4). In the Tanawal area, the maximum FL value was documented for *Momordica charantia, Acacia modesta, Citrullus lanatus* and *Tamarindus indica* (95.2 % for each). The minimum FL values were documented for *Diospyros lotus, Crataegus songarica* and *Rydingia limbate*. It reflected their significance for their particular uses. The plants with highest FL were considered the most useful [36]. The most common species, with FL exceeding 85% were *Cleome brachycarpa*, *Withania somnifera*, *Indigofera argentea*, *Euphorbia granulate* and *Chrozophora plicata*. The high FL values may be attributed to the extensive traditional knowledge and experience of the local community regarding the specific therapeutic properties of these plants. Local communities often develop a deep understanding of the medicinal uses of plants through trial and error, observations, and knowledge passed down through generations. The effectiveness of these plants in treating specific ailments may have been consistently observed over time, contributing to their high FL values.

### Relative Popularity level (RPL)

In our study, its value ranged from 0.17 to 0.38. the highest value was calculated for *O. linifolia* (0.38) commonly used to cure diarrhea. Some other plants with high RPL values were *D. bipinnata, E. crassipes* and *M. Africana* (0.36 for each). Some plants were reported with low RPL values i.e., *H. europaeum, S. conoidea, A. indicum, S. oleoides* (0.20) and *S. virginianum* (0.18). The lowest RPL value (0.17) was calculated for *M. azedarach* (Table 4). In a previous study, the highest value was obtained for *Anagallis arvensis, Citrullus colocynthis, Ficus variegate* and *Ocimum bacilicum* (1.0 for each)*.* Some other plants with higher RPL values in the study area were *Ranunculus laetus* (0.9), *Malva parviflora* (0.8) and *Lythrus aphaca* (0.7). Plants with lower RPL values were *Rumex dentatus* (0.2) and *Saccharum spontaneum* (0.3) [37]. The higher RPL values for these plants may be attributed to several factors. First, these plants might have distinct pharmacological properties that make them particularly effective in treating prevalent health conditions in community. Additionally, cultural practices, historical use and local knowledge could contribute to the popularity of these plants, as communities often rely on traditional remedies that have been passed down through generations.

### Rank Order Priority (ROP)

In the current study, *O. linifolia* (19.0) was ranked at the top used for diarrhea and the lowest value was reported for *M. azedarach* (7.61) for skin diseases. *O. linifolia* was followed by *D. bipinnata* (17.18), *E. crassipes* (17.11), *M. africana, L. perenne* (16 for each), *B. diffusa* (13.04), *S. vera, G. lotoides* (12.38), *H. curassavicum*, *M. albus* (12.27), *M. sylvestris* (12.0), *O. corniculata, C. brachycarpa* (11.99), *C. jwarancusa, C. album* (9.99 for each), *H. europaeum* (9.69) and so on. Lowest value (7.61) was calculated for *M. azedarach* (Table 4). In a survey conducted in District Hafizabad, an ethnobotanical study included a quantitative analysis known as Rank Order Priority to assess the medicinal plants. This analysis calculated values for various plant species indicating their respective medicinal significance and popularity within the local community [38]. ROP has values ranging from 0.99 to 18.0. However, it varied in our study from 7.61 to 19.0. The minimum was calculated for the *Youngia japonica*. The maximum calculation was documented for *Ranunculus laetus* (18.0) and the minimum calculation was documented for *Euphorbia helioscopia* (3.33), *Citrullus colocynthus* (6.66), *Cornopus didymus* (1.66), *Carssia opaca* (0.29), *Dicanthium annulatum* (5.99), *Eclipta prostrate* (3.99) and *Fumaria indica* (2.0) [39]. The higher ROP values calculated for specific plants such as *O. linifolia, D. bipinnata, E. crassipes* and* M. africana* in our study may be attributed to their perceived efficacy and prevalence in treating prevalent health issues like diarrhea. These plants likely hold cultural significance and have been traditionally recognized for their medicinal properties leading to their elevated ROP values. Comparisons with the available literature, such as studies from District Hafizabad, suggest that regional variations in plant prioritization exist due to differences in local knowledge, cultural practices and the prevalence of specific health concerns. The higher ROP values for certain plants may stem from their historical use, community consensus and observed effectiveness contributing to their prioritization in traditional medicinal practices.

### Frequency index (FI)

The maximum FI value in our study was documented for *C. melo* (3.22) followed by *P. glabra*, *P. barbata, L. pyrotechnica* (3.024 for each), *A. indicum, S. virginianum, C. jwarancusa, C. brachycarpa, C. conglomeratus, C. album, H. europaeum* (2.82 for each), *D. metel, D. muricata, S. vera, A. tenuifloius, G. lotoides, C. spinosa*, *S. conoidea, C. oxyacantha* (2.621 for each), *M. azedarach, N. plumbaginifolia* (2.41 for each), *F. pulverulenta, S. oleoides, D. bipinnata, T. resupinatum, P. harmala, L. perenne, M. sylvestris* (2.01 for each), *E. crassipes* (1.81) and the lowest value (1.61) was calculated for two plants *O. linifolia* and *A. lagopoides* (Table 4). In an ethnobotanical survey in Nepal, the frequency index of medicinal plants was calculated and it determined and it varied from 1.23 to 86.41. The highest value was documented for *Ricinus communis* (86.41) and the minimum value was documented for *Citrus limon, Camellia sinensis, Artocarpus lakoocha* and *Dolichos lablab* (1.22 for each) [40]. *Achyranthes aspera* has a frequency index of 14.81, *Centella asiatica* (30.86), *Dioscorea bulbifera* (74), *Mimosa pudica* (13.58) and *Jatropha curcas* (7.40) are some of the other species used in the study region. FI values calculated for specific plants in our study, such as *C. melo, P. glabra, P. barbata* and* L. pyrotechnica* could be attributed to several factors that make these plants more prevalent in traditional medicinal practices. These plants might possess distinct therapeutic properties that are highly effective in treating various health conditions leading to their frequent utilization by the local community. Additionally, cultural preferences, historical practices and indigenous knowledge may contribute to the popularity of these plants. The availability and accessibility of these plants in the local environment could also play a crucial role in their frequent use. Comparisons with the ethnobotanical survey in Nepal indicate that the context, cultural beliefs and ecosystem diversity influence the FI values supporting the idea that plant usage patterns are region-specific. Moreover, the consistency of plant application in traditional medicine as reflected by higher FI values may establish a stronger cultural connection reinforcing the continued use of these plants in the community.

### Cultural significance index (CSI)

The CSI value in our investigation varied from 0.5 to 4.0. The maximum value was documented for *C. melo* (4.0) and it was followed by *P. glabra, L. pyrotechnica* (3.7 for each), *A. indicum, S. virginianum, C. jwarancusa, C. brachycarpa, C. conglomeratus, S. oleoides, H. europaeum* (3.5 for each), *D. metel, D. muricata, S. vera, A. tenuifloius, G. lotoides, A. graveolens, D. bipinnata, T. resupinatum, H. curassavicum, M. albus* (2.7 for each), *F. benghalensis, C. gigantea, M. africana, P. aculeata, P. harmala, M. sylvestris* (2.5 for each), *P. barbata* (1.8), *L. perenne* (1.2), *E. crassipes* (1.1) and the lowest value (0.5) was calculated for *O. linifolia, A. lagopoides* (Table 4). The CSI value is based on how well and how intensely a species is used to treat a disease [22]. A study from KPK, Pakistan reported the highest known CSI for *Lawsonia inermis*, *Piper nigrum*, *Plantago major* and *Senna alexandrina* [41]. The CSI values may be influenced by various factors contributing to their cultural significance. These factors could include historical use, traditional knowledge passed down through generations, symbolic importance and effectiveness in treating prevalent health issues in community. In many cultures, certain plants have been integral to traditional healing practices for centuries, contributing to their higher CSI values. Additionally, perceived efficacy of these plants in treating a range of ailments may enhance their cultural significance. It's crucial to recognize that cultural values associated with plants can vary across regions and communities explaining differences observed in comparison with other studies. Local beliefs, folklore and historical practices all contribute to the perceived cultural significance of plants.

### Novelty index

**Fig. 8:**
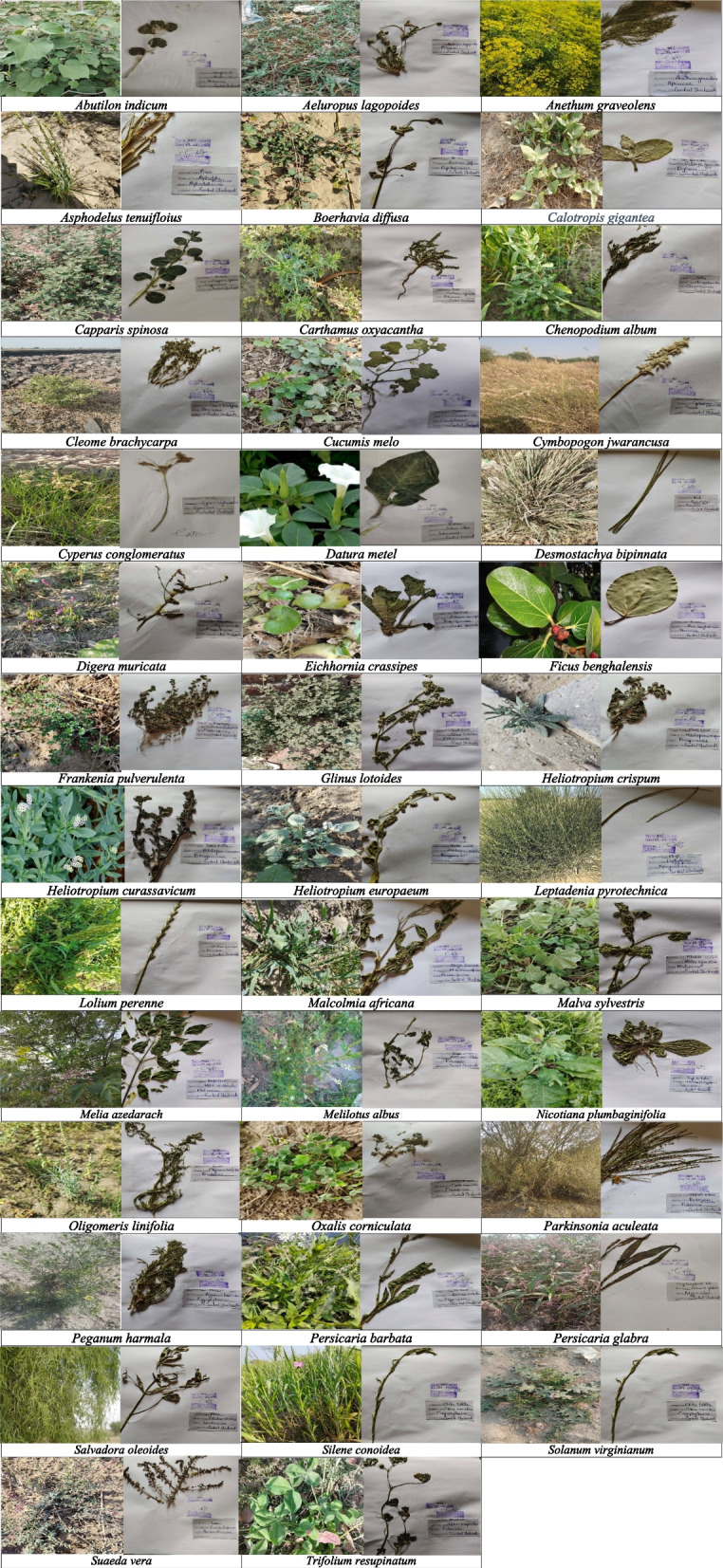
Ethnobotanical plants of the study area

**Fig. 9:**
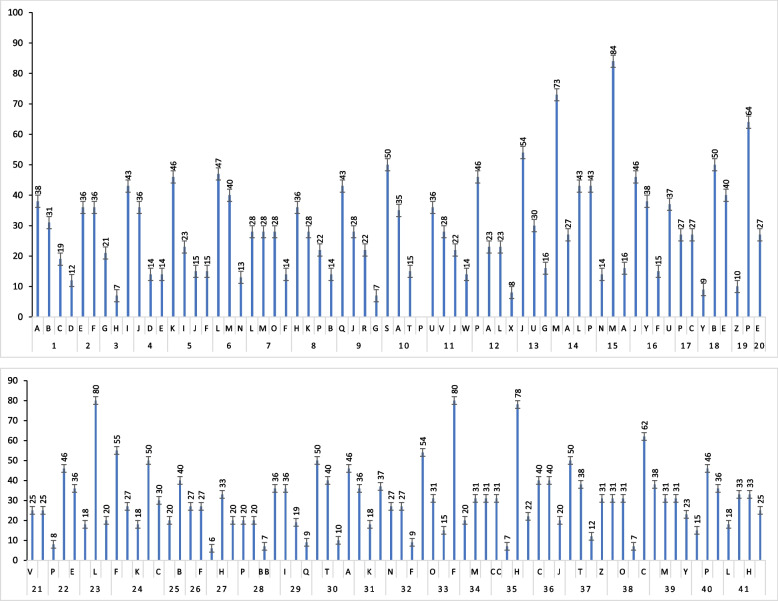
Percentage usage of ethnomedicinal plants to cure different ailments. A. Inflammation, B. Diarrhea, C. Cancer, D. Piles, E. Wound healing, F. Diabetes, G. Stomach, H. Ulcer, I. Eye diseases, J. Diuretic, K. Jaundice, L. Cough, M. Asthma, N. Fever, O. Pain, P. Skin diseases, Q. Laxative, Hepatitis, S. Anti-microbial, T. Rheumatism, U. Constipation, V. Kidney diseases, W. Cholesterol, X. Gargles, Y. Gonorrhea, Z. Analgesic., AA. Cardiac diseases, BB. Blood purifies, CC. Astringent, DD. Snake bite. 1. *Zygophyllum arabicum*, 2. *Aeluropus lagopoides*, *Anethum graveolens*, 4. *Asphodelus tenuifloius*, 5*. Boerhavia diffusa*, 6. *Calotropis gigantea*, 7. *Capparis spinosa*, 8. *Carthamus oxyacantha*, 9. *Chenopodium album*. 10. *Cleome brachycarpa*, 11. *Cucumis melo*, 12. *Cymbopogon jwarancusa*, 13. *Cyperus conglomeratus*, 14. *Datura metel*, 15. D*esmostachya bipinnata,* 16*. Digera muricata,* 17*. Eichhornia crassipes*, 18*. Ficus benghalensis, 19. Frankenia pulverulenta, 20. Glinus lotoides.*21. *Heliotropium crispum*, 22. *Heliotropium curassavicum*, 23. *Heliotropium europaeum*, 24. *Leptadenia pyrotechnica*, 25. *Lolium perenne*, 26. *Malcolmia africana*, 27. *Malva sylvestris*, 28. *Melia azedarach*, 29. *Melilotus albus*, 30. *Nicotiana plumbaginifolia*, 31. *Oligomeris linifolia*, 32. *Oxalis corniculata*, 33*. Parkinsonia aculeata*, 34. *Peganum harmala*. 35.*Persicaria barbata*, 36. *Persicaria glabra,* 37. *Salvadora oleoides,* 39. *Solanum virginianum* 40. *Suaeda vera*, 41. *Trifolium resupinatum*

Upon comparing ethnomedicinal data collected from the study area with information from 30 other studies, distinct and novel uses of some plant species were showed. *L. perenne* was reported as a novel species in the study area. The application of fresh paste from *A. lagopoides* for wound healing, a species rarely documented in other regions, was first reported in our study conducted in Dunyapur. Additionally, herbal tea made from this plant was found to be effective for treating diarrhea. *F. pulverulenta* identified in the study exhibited analgesic properties for skin diseases. Similarly, *M. africana*, a plant not previously documented in the study area previously is reported and its flower, leaves and seed are utilized for treating cardiac diseases and diabetes (refer to Figs. 8 and 9). In contrast, prior studies indicated use of *Abutilon indicum* for the treatment of fever, inflammation, ulcers, gonorrhea, and chest infections [42]. our current study reported that this plant was found to be effective in treating piles. *S. virginianum* was extensively utilized by the local population in the study area. Upon reviewing previous literature, it was discovered that this plant had been traditionally employed for asthma, pain, and as a diuretic [43]. However, our current study revealed a novel use, indicating that it was employed in the treatment of liver problems in the study area*.* In earlier literature, it was documented that *Ficus benghalensis* had been utilized for treating gonorrhea, chronic cough, flu and influenza [44]. However, our current study introduced a novel application, revealing its use in wound healing which had not been explored in previous literature. *S. oleoides* was traditionally known for its efficacy in addressing toothache mouthwash, gonorrhea, spleen issues, ulcers, epilepsy and cholesterol. Notably, our study showed a new application highlighting its effectiveness as an analgesic among the local residents with a decoction made from its leaves being utilized for this purpose. While *S. conoidea* had been previously acknowledged for wound healing, emollient properties and as a fumigant, deemed beneficial for ophthalmic problems, our current study reported its rare use in cancer cases. Another plant species, *M. albus*, had been recognized in prior research for treating sore eyes, conjunctivitis, earache, colic flatulence and as an eyesight tonic [45]. However, our study presented a novel application, indicating its use as a laxative in the study area. In a study from Cholistan, *C. conglomeratus* was acknowledged for addressing hyperacidity, constipation and indigestion [46], whereas our study reported its application for stomach pain (Table 4). The novelty index in enhance the understanding of unique applications of medicinal plants across the study area.

### Jaccard index (JI)

Comparison with existing literature involved examining data from studies spanning the years 2006 to 2023 in the research region (Table 5). The overlap in species between the study area and its neighboring areas ranged from 0 to 15 with Bahawalpur recording the highest number of shared species (15). The Cholistan Desert exhibited a maximum JI value at 12.60 while Deosai National Park reported the lowest (JI=0.00). Internationally, Saudi Arabia demonstrated the highest JI at 4.20. These comparative analyses provide insights into the ethnobotanical diversity and similarities among different regions contributing valuable information to the existing body of knowledge. The JI values calculated for the comparison of the present work with previous studies reflect the extent of similarity in medicinal plant species composition across different regions. The varying JI values can be attributed to several factors influencing plant diversity and traditional knowledge, including geographical, environmental, and cultural aspects. Firstly, the higher JI values, such as the one reported for the Cholistan Desert (JI=12.60) suggest a significant overlap in medicinal plant species between the study area and that specific region. This could be due to similar ecological conditions, shared plant resources or cultural exchanges that have contributed to a common pool of traditional knowledge. Conversely, the lowest JI values, like the one recorded for Deosai National Park (JI=0.00) indicate a complete dissimilarity in medicinal plant species. This lack of overlap could be attributed to distinct environmental conditions, different ecological zones or unique cultural practices that influence the selection and use of medicinal plants. Comparisons with studies in Hafizabad and other regions revealed varying degrees of commonality. The number of shared species and the resulting JI values highlight the regional specificity of ethnobotanical knowledge. Similarities may arise from historical connections, migration patterns or shared ecological features while differences influenced by local preferences, climate variations or unique cultural practices.

**Table 5:** Jaccard Index (JI)

**Sr.**	**Citations**	**Area**	**SY**	**TRSs**	**NPSU**	**NPDU**	**TSCBA**	**PPSU**	**PPDU**	**JI**
**Comparison with studies from neighboring areas of Dunyapur, Punjab, Pakistan**										
1		Dunyapur, Lodhan [47]	2017	62	0	2	2	0.00	3.22	1.98
2		Bahawalpur [26]	2014	123	15	0	15	12.19	0.00	10.07
3		Multan [48]	2014	44	3	3	6	6.81	6.81	7.59
4		Cholistan Desert [8]	2023	93	10	5	15	10.75	5.37	12.60
**Comparison with studies of other areas of Punjab, Pakistan**										
5		Thal, Layyah [40]	2015	78	5	4	9	6.41	5.12	8.18
6		Isakhel, Mianwali [25]	2006	55	4	5	9	7.27	9.09	10.34
7		Southern Punjab [6]	2021	58	5	1	6	8.62	1.72	6.45
8		Central Punjab [41]	2013	102	5	8	13	4.90	7.84	10
9		Tehsil Gojra [30]	2020	47	2	1	3	6.38	2.13	3.53
10		Hafizabad [16]	2017	85	5	2	7	5.88	2.35	5.88
11		Navapind, Sheikhupura [49]	2017	96	3	1	4	3.12	1.04	3.01
12		Miani Sahib, Lahore [20]	2016	75	3	4	7	4	5.33	6.42
13		Kasur [1]	2020	78	0	3	3	0.00	3.85	2.59
15		Kharian, Gujrat [50]	2021	49	4	2	6	8.16	4.08	7.14
16		Pind Dadan Khan [32]	2011	69	10	0	10	14.49	0.00	10
**Comparison with studies from some other areas outside of Punjab, Pakistan**										
18		Tanawal, Western Himalyas [34]	2022	66	0	1	1	0.00	1.5	0.94
19		Charhoi Kotli, AJK [4]	2021	100	6	2	8	6	2	6.01
20		Deosai National Park, GB [28]	2021	47	0	0	0	0.00	0.00	0.00
21		Dir Kohistan Valley [51]	2008	34	2	0	2	5.88	0.00	2.73
**Comparison with studies outside of Pakistan**										
22		Odisha, India [52]	2021	85	1	3	4	1.17	3.53	3.28
23		Tibet, China [44]	2022	121	0	0	0	0.00	0.00	0.00
24		Iran [53]	2023	174	0	2	2	0.00	1.15	0.93
25		Saudi Arabia [42]	2013	83	4	1	5	4.81	1.20	4.20
26		Tuscany, Italy [43]	2023	78	0	1	1	0.00	1.28	0.84
27		Sri Lanka [54]	2021	133	0	2	2	0.00	1.50	1.16
28		Nepal [55]	2020	215	3	2	5	1.39	0.93	1.99
29		Ethiopia [11]	2017	135	0	1	2	0.00	0.74	1.33
30		Morocco [2]	2022	280	1	1	2	0.35	0.35	0.62

Key: *SY* Study area, *TRSS* Total number of reported species, *NPSU* Several plants with similar uses, *NPDU* Several plants with dissimilar uses, *TSCBA* Total number of species common in both areas, *PPSU* Percentage of plants with similar uses, *PPDU* Percentage of plants with dissimilar uses, *JI* Jaccard

## Conclusions

This ethnobotanical exploration in Dunyapur, Pakistan provides a nuanced understanding of traditional medicinal practices deeply embedded in the local culture. The documentation of 41 plant species across 28 families sheds light on biodiversity and intricate relationships between communities and their natural surroundings. The findings, encompassing various indices and values highlight importance of certain plants in addressing prevalent health concerns and highlight their cultural significance. The study not only enriches repository of traditional knowledge but also emphasizes dynamic nature of these practices continuously evolving to adapt to changing contexts. Furthermore, the seamless integration of food perceptions, health practices and sustainable choices highlights holistic well-being ingrained in age-old indigenous wisdom of Dunyapur, reaffirming the enduring relevance of traditional medicine in this community. Further interdisciplinary research and community engagement are encouraged to sustain and promote the rich traditional knowledge while exploring potential applications in modern healthcare practices. Additionally, initiatives for conservation of biodiversity and creation of awareness programs can contribute to the preservation of this valuable traditional knowledge.

### Supplementary Information

**Additional file 1**.

## Data Availability

The original data is presented in the article. There is no supplementary data.
