# Supplementary material for: Exploring the benefits of wild plants in dietary nutrition: investigating perspectives, choices, health impacts and sustainable practices
Source: BMC Complement Med Ther. 2024 Feb 14;24:86. doi: 10.1186/s12906-024-04379-4 (PMC10865668; doi:10.1186/s12906-024-04379-4)
Supplement: Supplementary file 1 — Additional file 1. [file 12906_2024_4379_MOESM1_ESM.docx]

**QUESTIONNAIRE FOR ETHNOMEDICINAL DATA COLLECTION**

**Serial No**. ……………… **Date** ……………………

1. **Informant’ details**

| Name…………………. | Occupation………………... |
| --- | --- |
| AGE………………...... | Gender……………………. |
| Educational status….... | Location…………………... |

1. **Plant’s information**
2. Vernacular name ……………………………………………………………………
3. Used as ……………………………………………………………………………...
4. Type of plant

(a) wild (b) cultivated (c) imported

1. Use of plant

(a) medicinal (b) cosmetic (c) other

1. Harvesting technique

(a) manual (b) mechanical (c) other

1. Plant state

(a) Fresh (b) Dried (c) both

1. Preparation mode

(a) infusion (b) decoction (c) powder (d) herbal tea (e) juice (f) paste

(g) others

1. Administration mode
2. oral (b) massage (c) topical
3. Dosage: number of doses taken a day

(a) 1 time/day (b) 2 time/day (c) 3 time/day

1. Duration of use

(a) one day (b) one week (c) one month (d) until healing

1. Collection period

(a) summer (b) winter (c) autumn (d) spring (e) all-year record

1. Which part of the plant is used as a medicine:

(a) flowers (b) stem (c) seed (d) bark (e) leaves (f) root (g) fruit (h) whole plant

1. Dose used

(a) pinch (b) spoon (c) other

1. Preservation method

(a) protect from light (b) exposed to light (c) other

1. How did you obtain the knowledge of medicinal plants?

(a) from elders (b) formal training (c) other

17. Changes in abundance of the plant for the last 10 years

a) More abundant b) Rare c) Same

18. Where do you get the plant?

(a) Garden (b) Field (c) Forest (d) Other

19. Demand for this plant

1. Increase b) Decrease

20. How frequently have you used medicinal plants over the last 12 months?

a) Daily b) Sometimes c) When unwell d) Never

21. How would you describe your health?

a) Excellent b) Poor c) Very good d) Good e) Not good

22. Where do I find more information about traditional medicinal plants?

1. Hakims (b) traditional markets (c) local communities (d) others

23. Success rate of treatment of this plant

(a) high (b) low (c) average (d) none

24. Reason of the plant for considered as medicines

1. Religious belief b) Traditional (c)Personal experience d) other

25. Is the plant used together with other plants? (a) yes (b) no

26. Is it sold in the local market? (a) yes (b) no

27. Have you used this plant for treatment? (a) yes (b) no

28. Is this plant used as food? (a) yes (b) no

1. Is this plant an alternative to allopathic medicines? (a) yes (b)no
2. Is this plant easily available? (a) yes (b) no
3. Is this plant toxic? (a) yes (b) no
4. Are you interested in traditional recipes? (a) yes (b) no
5. Do you use this remedy for children? (a) yes (b) no
6. Are the herbal supplements safe to use? (a) yes (b) no
7. Are these traditional methods bringing results? (a) yes (b) no
8. Does it have any side effects? (a) yes (b) no
9. Are there any precautions when using the remedy? (a) yes (b) no
10. Conservation of medicinal plants is necessary? (a) yes (b) no
11. Do you inform the health professional (physician) during? (a) yes (b) no
12. In the clinical interview that you using herbal medicines? (a) yes (b) no
